# CD8^+^ TILs in NSCLC differentiate into TEMRA via a bifurcated trajectory: deciphering immunogenicity of tumor antigens

**DOI:** 10.1136/jitc-2021-002709

**Published:** 2021-09-30

**Authors:** Sung-Woo Lee, He Yun Choi, Gil-Woo Lee, Therasa Kim, Hyun-Ju Cho, In-Jae Oh, Sang Yun Song, Deok Hwan Yang, Jae-Ho Cho

**Affiliations:** 1 Division of Integrative Biosciences and Biotechnology, Pohang University of Science and Technology, Pohang, Gyeongsangbukdo, Republic of Korea; 2 Department of Internal Medicine, Chonnam National University Medical School, Hwasun Hospital, Hwasunup, Jeollanamdo, Republic of Korea; 3 Department of Thoracic and Cardiovascular Surgery, Chonnam National University Medical School, Hwasun Hospital, Hwasunup, Jeollanamdo, Republic of Korea; 4 Department of Microbiology and Immunology, Chonnam National University Medical School, Hwasunup, Jeollanamdo, Republic of Korea; 5 Medical Research Center for Combinatorial Tumor Immunotherapy, Chonnam National University Medical School, Hwasunup, Jeollanamdo, Republic of Korea; 6 Immunotherapy Innovation Center, Chonnam National University Medical School, Hwasunup, Jeollanamdo, Republic of Korea; 7 BioMedical Sciences Graduate Program, Chonnam National University Medical School, Hwasunup, Jeollanamdo, Republic of Korea

**Keywords:** biomarkers, tumor, lymphocytes, tumor-infiltrating, CD8-positive T-lymphocytes, immunotherapy

## Abstract

**Background:**

CD8^+^ tumor-infiltrating lymphocytes (TILs) comprise phenotypically and functionally heterogeneous subpopulations. Of these, effector memory CD45RA re-expressing CD8^+^ T cells (Temra) have been discovered and characterized as the most terminally differentiated subset. However, their exact ontogeny and physiological importance in association with tumor progression remain poorly understood.

**Methods:**

We analyzed primary tumors and peripheral blood samples from 26 patients with non-small cell lung cancer and analyzed their phenotypes and functional characteristics using flow cytometry, RNA-sequencing, and bioinformatics.

**Results:**

We found that tumor-infiltrating Temra (tilTemra) cells largely differ from peripheral blood Temra (pTemra), with distinct transcriptomes and functional properties. Notably, although majority of the pTemra was CD27^−^CD28^−^ double-negative (DN), a large fraction of tilTemra population was CD27^+^CD28^+^ double-positive (DP), a characteristic of early-stage, less differentiated effector cells. Trajectory analysis revealed that CD8^+^ TILs undergo a divergent sequence of events for differentiation into either DP or DN tilTemra. Such a differentiation toward DP tilTemra relied on persistent expression of CD27 and CD28 and was associated with weak T cell receptor engagement. Thus, a higher proportion of DP Temra was correlated with lower immunogenicity of tumor antigens and consequently lower accumulation of CD8^+^ TILs.

**Conclusions:**

These data suggest a complex interplay between CD8^+^ T cells and tumors and define DP Temra as a unique subset of tumor-specific CD8^+^ TILs that are produced in patients with relatively low immunogenic cancer types, predicting immunogenicity of tumor antigens and CD8^+^ TIL counts, a reliable biomarker for successful cancer immunotherapy.

## Background

Phenotypic analyses have been extensively performed with humans to distinguish functionally and phenotypically different subsets of CD8^+^ T cells.^1–3^ Several surface markers that discriminate CD8^+^ T cell subsets have been discovered, including C-C chemokine receptor type 7 (CCR7), CD45RA, CD45RO, and CD57.^2^ Particularly, CCR7 and CD45RA have been widely used for separating distinct differentiation states of human CD8^+^ T cells in both healthy and disease conditions.^4^ Fully matured CD8^+^ T cells exit the thymus as naïve T cells (CCR7^+^CD45RA^+^; Tn).^5^ On activation, Tn cells show a downregulated expression of CCR7 and CD45RA and differentiate into effector T cells (CCR7^−^CD45RA^−^; Teff).^6^ Teff cells further differentiate into either effector memory (CCR7^−^CD45RA^−^; Tem) or central memory T cells with upregulation of CCR7 (CCR7^+^CD45RA^−^; Tcm). However, in some cases, for which exact mechanisms remain largely unknown, such cells re-express CD45RA to become effector memory CD45RA re-expressing T cells (CCR7^−^CD45RA^+^; Temra).^2^

Among these subsets of human CD8^+^ T cells, Temra is considered a terminally differentiated subset and exhibits low proliferation capacity and differentiation plasticity, while preserving increased production of perforin and granzyme B.^2 3 7–10^ With regard to the differentiation path, Temra cells show short telomere length and a low number of T cell receptor (TCR) excision circles,^9 11^ indicating that Teff and/or Tem cells need to undergo repetitive proliferation for transitioning into Temra cells. However, the mechanisms underlying the regulation of such transitory events and the coupling of recurrent proliferations with the critical stage of the re-expression of CD45RA, a hallmark of Temra, remain poorly understood.

Although Temra is considered the most terminally differentiated subset, complex variables involved in past immune responses allow some degrees of heterogeneity. In this regard, costimulatory molecules CD27 and CD28 are often used to further define human CD8^+^ T cell differentiation in depth.^1 11^ Tn cells show a homogeneously high expression of both CD27 and CD28 (CD27^+^CD28^+^); however, Teff/Tem and Temra cells exhibit variable levels of expression of these molecules.^1 11^ Although high expression levels of either of these markers are not common in Temra,^1 11^ several studies have demonstrated Temra cells expressing CD27 and/or CD28.^1 11 12^ CD27^+^CD28^+^ Temra cells show low expression levels of KLRG1 and CD57 and demonstrate a relatively less differentiated state compared with CD27^−^CD28^−^ Temra cells.^1^ However, lineage relationship between CD27^+^CD28^+^ and CD27^−^CD28^−^ Temra cells and their physiological relevance in the association of particular immune contexts with diseases such as cancers remain largely elusive.

Compelling evidence has been demonstrated highlighting the role of CD8^+^ tumor-infiltrating lymphocytes (TILs) in cancer immunotherapy.^13 14^ Since the majority of CD8^+^ TILs have activated/memory phenotypes, many studies have focused on the Teff/Tem population by analyzing various costimulatory and/or coinhibitory markers to further characterize these cells into discrete subpopulations and understand their roles in tumor immunity.^13–15^ Temra cell population, commonly found in peripheral blood mononuclear cells (PBMCs), has also been reported to exist in CD8^+^ TILs in similar proportions^16^; however, its development and physiological importance in relation to tumors remain unclear.

In this study, we analyzed tumor and peripheral blood Temra (pTemra) from patients with non-small cell lung cancer (NSCLC) to define their phenotypic and functional characteristics and physiological relationship with tumor progression. Tumor-infiltrating Temra (tilTemra) exhibited functionally and transcriptionally distinct phenotypes compared with pTemra. In particular, Temra cells were derived from distinct Teff/Tem cells by bifurcating into two different subsets, namely CD27^+^CD28^+^ and CD27^−^CD28^−^. Importantly, the CD27^+^CD28^+^ phenotype was more frequently—though variable for each patient with NSCLC—observed in tilTemra, and its presence was largely dependent on a persistent low-grade TCR signal, implying a close relationship with the immunogenicity of tumor antigens. Accordingly, we demonstrated that the relative proportion of CD27^+^CD28^+^ Temra inversely correlates with the number of CD8^+^ TILs and tumor mutation burden (TMB), known to affect the generation of neoantigens and the number of tumor-reactive CD8^+^ TILs. Together, these findings strongly suggest that CD27^+^CD28^+^ Temra cells reflect a unique ‘prequel’ of tumor-specific CD8^+^ T cell responses and can predict tumor prognosis and therapeutic efficacy of patients with cancer undergoing immunotherapy.

## Methods

### Human samples

For the purpose of this study, tumor tissues and blood samples from patients with NSCLC (n=26) were harvest at Chonnam National University Hwasun Hospital (Korea). In some studies, another set of blood samples from patients with a terminal stage of NSCLC (n=30) were used. The clinical characteristics of all patients in two cohorts are summarized in online supplemental tables 1 and 2.

10.1136/jitc-2021-002709.supp1 Supplementary data

### Sample preparation

Tumor tissues were received directly from the operating room and processed immediately. Tissues were chopped into small pieces, then digested with 0.5 mg/mL Collagenase IV (Gibco) and 200 U DNase I (Roche) in a 37°C magnetic stirrer. TILs were purified from the digested tissues using Percoll (Cytiva). Blood samples were harvested in BD Vacutainer (BD Biosciences). PBMCs were purified using Lymphoprep (Alere Technologies).

### Flow cytometry

Cell suspensions of TILs or PBMCs were prepared and stained for FACS analysis with the following antibodies (purchased from BioLegend, eBioscience, and BD Biosciences): CD45RA (HI100), CD45RO (UCHL1), CD27 (LG.7F9), CD28 (CD28.2), CD57 (QA17A4), CD5 (L17F12), CD3 (HIT3a), CCR7 (G043H7), CD8 (SK1), CD279 (MIH4), Perforin (B-D48), Granzyme B (GB11), IFNg (B27), IL2 (MQ1-17H12), and Ki67 (Ki-67). Flow cytometry samples were run using FACSCantoII (BD Biosciences) or CytoFLEX LX (Beckman Coulter) and analyzed by FlowJo software (Tree star).

### Intracellular staining

Cells were plated in 96-well cell culture plate (1×10^6^ cells/well) and cultured for 4 hours with eBioscience Cell Stimulation Cocktail (plus protein transport inhibitors) (Invitrogen) in 37°C CO_2_ incubator. Cells were stained for surface markers, then fixed and permeabilized using BD Cytofix/Cytoperm buffer (BD Biosciences). Cells were then stained for indicated intracellular molecule and analyzed by flow cytometry. For Ki-67 staining, eBioscience Foxp3/Transcription Factor Staining Buffer Set (eBioscience) was used with ex vivo samples.

### Relative telomere length

Relative telomere length was measured using relative telomere to single copy ratio as previously described.^17^ In brief, 1×10^5^ cells were purified and used for genomic DNA extraction with Genomic DNA Extraction Kit (Bioneer) according to the manufacturer’s instruction. Extracted DNA (20 ng/sample) was used for quantitative PCR with SYBR Green qPCR Master Mix (Maxima) with following primers:

tel1: GGTTTTTGAGGGTGAGGGTGAGGGTGAGGGTGAGGGT

tel2: TCCCGACTATCCCTATCCCTATCCCTATCCCTATCCCTA

36B4u: CAGCAAGTGGGAAGGTGTAATCC

36B4d: CCCATTCTATCATCAACGGGTACAA

### In vitro activation

Indicated cell types were purified using FACSAria III (BD Biosciences) and plated (5×10^3^ cells/well) in anti-CD3 and anti-CD28 coated Nunc MaxiSorp Flat-Bottom Plate (Invitrogen) or 96-well cell culture plate (SPL Life Sciences) for TCR or cytokine stimulation, respectively. For some experiments, cells were labeled with 2.5 µM CellTrace Violet (Invitrogen) (CTV) prior to stimulation. 0.5 ng/mL of interleukin 7 (IL-7) was subjected to every well to promote survival. Cells were stimulated with indicated stimulations for 7 days, then stained and analyzed by flow cytometry.

### Bioinformatics

For RNA-sequencing (RNA-seq), 3×10^5^ cells were purified and used for RNA extraction with TriZOL (Invitrogen). Library was constructed using TruSeq RNA Sample Prep Kit (Illumina) and sequenced with an Illumina HiSeq2500 sequencer (Illumina) according to manufacturer’s protocols. Principal component analysis and hierarchical clustering were performed using Factoextra R Package. t-distributed stochastic neighbor embedding (tSNE) analysis was performed using FlowJo Software (Tree star). CD3^+^CD8^+^ cells were first gated, then used for tSNE analysis with following parameters: CD27, CD28, CD45RA, CD45RO, CD8, CD3, CD5, CCR7, Perforin, and CD57. FLOW-MAP was performed using FLOWMAPR R Package according to developer’s protocol.^18^ In brief, CD3^+^ CD8^+^ cells were gated and exported using FlowJo. Exported fcs file was used for FLOW-MAP. Force-directed layout (Force Atlas) was carried out with Gephi software. Gene set enrichment analysis (GSEA) was carried out by GSEA tool (Broad Institute). Following options were selected for the analysis: Number of permutations, 1000; Collapse dataset to gene symbols, false; Permutation type, phenotype; Enrichment statistic, weighted; Metric for ranking genes, Signal2Noise. For sample distance matrix, PBMCs and TILs of 6 patients with NSCLC were used to analyze 8 TCR α/β usages (Va2, Vα7.2, Vα12.1, Vβ3, Vβ5b, Vβ8, Vβ12, and Vβ13.1) of peripheral or tumor infiltrated CD27^+/−^ Tem and CD27^+/−^ Temra cells. Sample distance matrix was performed using DESeq2 R Package.

### Statistics

Samples were tested for a normal distribution using normality tests (eg, Kolmogorov-Smirnov test). For normally distributed samples, paired or unpaired two-tailed Student’s t-test were performed. For samples that did not pass the normality tests, Mann-Whitney U test (for unpaired) or Wilcoxon matched-pairs signed rank test (for paired) were performed. The statistics used for each figure are indicated in the legends of the respective figure. All statistics were performed using Prism (GraphPad Software). Values of *p<0.05, **p<0.01, ***p<0.001, ****p<0.0001 were considered significant.

## Results

### CD8^+^ TILs contain Temra subset with more differentiated characteristics than Tem subset

**Figure 1:**
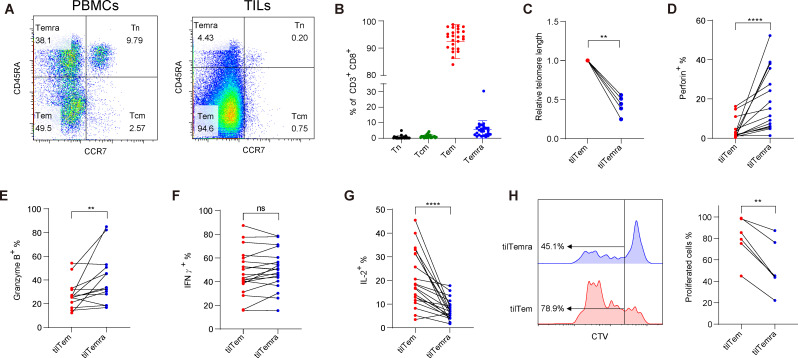
Characterization of CD8^+^ TILs derived from patients with NSCLC. (A) Representative flow cytometric data used for distinguishing CD8^+^ T cell subpopulations (Tn, Tcm, Tem, and Temra) from PBMCs (left) and TILs (right) using CCR7 and CD45RA. (B) Proportions of CD8^+^ T cell subpopulations in CD8^+^ TILs. (n=26) (C) Relative telomere lengths of tilTem and tilTemra. Telomere lengths were measured by the ratio of telomere and 36B4. Relative telomere lengths were calculated by dividing with the telomere lengths of tilTem obtained from the same tumor. (n=5) (D–G) TILs were restimulated for 4 hours and the frequencies of (D) Perforin, (E) Granzyme B, (F) IFN-γ, and (G) IL-2 producing tilTem or tilTemra were assessed by flow cytometry. (n=14–20) (H) tilTem and tilTemra were purified and labeled with CTV, then stimulated with anti-CD3 (5 µg/mL), anti-CD28 (2 µg/mL), and IL-2 (10 ng/mL) for 7 days. CTV dilution was assessed by flow cytometry and cells with diluted CTV were considered proliferated cells. (n=5) Statistical significance was performed with (C) Mann-Whitney U test, (D–G) Wilcoxon matched-pairs signed rank test, or (H) paired Student’s t-test. Values of *p<0.05, **p<0.01, ***p<0.001, ****p<0.0001 were considered significant. CCR7, C-C chemokine receptor type 7; CTV, CellTrace Violet; IFN-γ, interferon γ; IL-2, interleukin 2; NSCLC, non-small cell lung cancer; PBMCs, peripheral blood mononuclear cells; Tcm, central memory T cells; Tem, effector memory T cells; Temra, effector memory CD45RA re-expressing T cells; TILs, tumor-infiltrating lymphocytes; tilTemra, tumor-infiltrating Temra; Tn, naïve T cells.

To examine the heterogeneous CD8^+^ TILs, PBMCs and TILs from 26 patients with NSCLC were analyzed using flow cytometry. A summary of the clinical information of the patients is in online supplemental table 1. CD8^+^ T cells were separated into four different subpopulations based on the expression of CCR7 and CD45RA: CCR7^+^CD45RA^+^ Tn, CCR7^+^CD45RA^−^ Tcm, CCR7^−^CD45RA^−^ Teff/Tem (referred to as Tem), and CCR7^−^CD45RA^+^ Temra (figure 1A and online supplemental figure 1A). In contrast to PBMCs, Tn and Tcm cells were mostly undetectable in TILs (average <1%). Instead, TILs were primarily occupied by Tem, accounting for 68.2%–98.7% (average 92.29%; figure 1B and online supplemental figure 1B). Further, Temra cells were consistently found in TILs, accounting for 0.68%–30.4% (average 5.72%).

Temra is considered the most differentiated subset of human CD8^+^ T cells, as evidenced by the short length of telomeres and increased expression levels of perforin and granzyme B.^1 2 9^ Since most characterizations of Temra were previously established with pTemra cells, we examined whether tilTemra cells exhibit the standard characteristics of pTemra. Indeed, tilTemra demonstrated shorter telomeres than tilTem (figure 1C). In addition, tilTemra showed increased expression levels of perforin and granzyme B (figure 1D, E). However, IFN-γ production was similar among cells of both groups (figure 1F). Contrary to cytolytic functions, proliferation capacity decreased with T cell differentiation.^8 10 19^ Accordingly, tilTemra showed decreased IL-2 production compared with tilTem (figure 1G). A similar pattern was observed in their proliferation capacities on stimulation with anti-CD3/CD28 and IL-2 (figure 1H). These data suggest that tilTemra cells exhibit broadly similar characteristics to Temra cells, defined from PBMCs (online supplemental figure 1C–F).

### tilTemra has a unique transcriptome, different from pTemra

**Figure 2:**
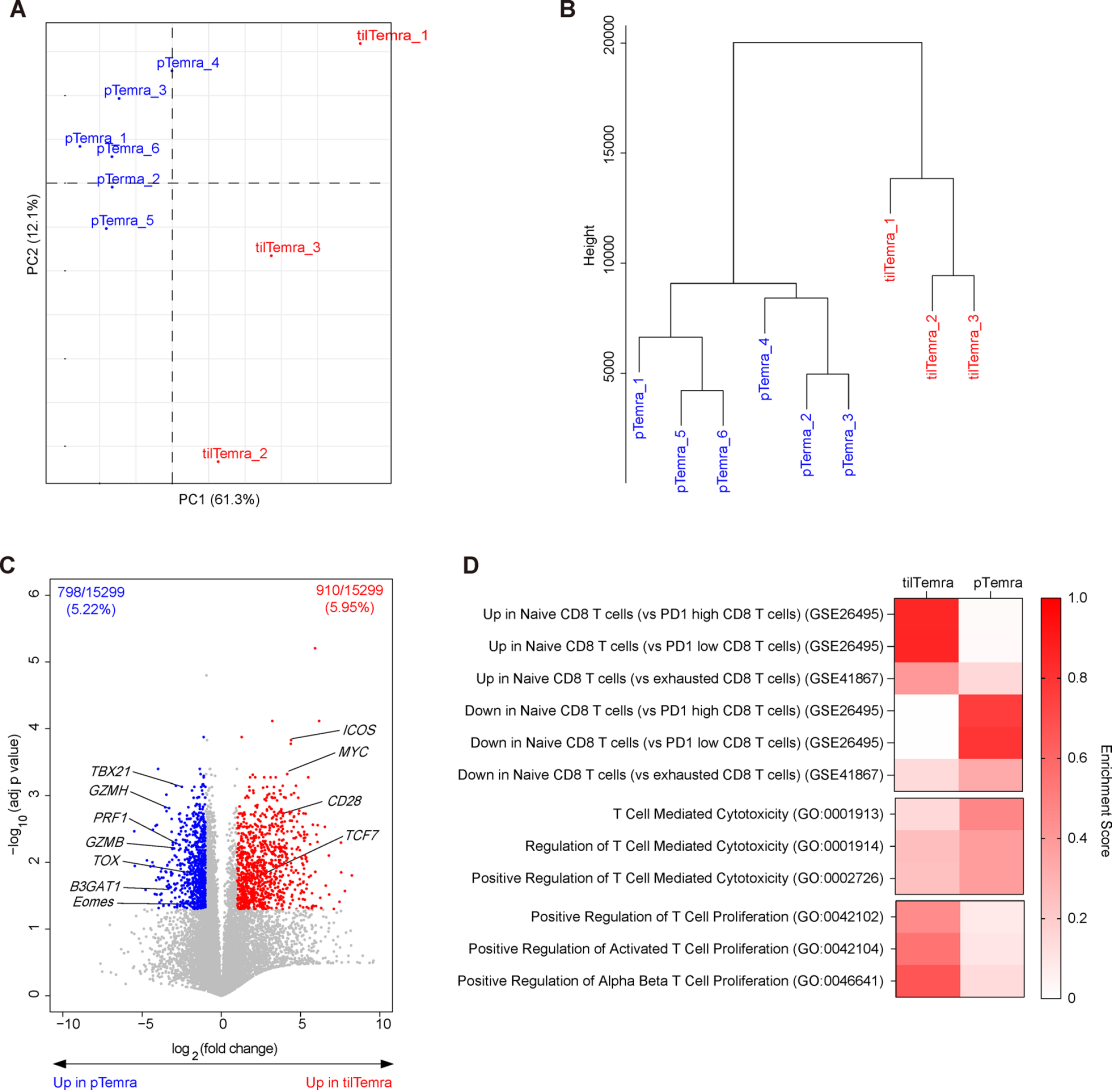
Comparison of transcriptome profiles between tilTerma and pTemra. (A–D) RNA seq data acquired from three tilTemra and six pTemra were used for transcriptome analysis. (A) Principal component analysis and (B) hierarchical clustering of tilTemra and pTemra. (C) A total of 15 299 genes with meaningful expression (FPKM >0) were used for generating volcano plot. P values were first calculated using t-test (two tailed) function in excel, then modified to adjusted p value (adj p value) using Benjamini-Hochberg procedure. Fold changes were calculated by dividing mean expression of pTemra from mean expression of tilTemra. Red dots (910 genes out of 15 299 genes; 5.95%) represent genes upregulated in tilTemra (fold change >2 and adj p<0.05) and blue dots (798 gene out of 15 299 genes; 5.22%) represent genes upregulated in pTemra (fold change <0.5 and adj p<0.05). (D) Summary of GSEA results using gene sets acquired from Broad Institute and Gene Ontology. Enrichment score represents absolute value of the highest or the lowest running Enrichment Score. pTemra, peripheral blood Temra; tilTerma, tumor-infiltrating Temra; FPKM, fragments per kilobase of exon per million; GSEA, gene set enrichment analysis.

Despite the overall similarity, we further investigated if tilTemra and pTemra were the same cellular population distributed to spatially different locations. For this, we compared the gene expression profiles of FACS-purified tilTemra and pTemra cells derived from patients with NSCLC (tilTemra_1–3, pTerma_1–3) and pTemra cells obtained from healthy donors (pTemra_4–6) by RNA-seq analysis. Principal component (PC) analysis showed clear separation of tilTemra from pTemra by the first PC (PC1) (figure 2A). The difference in the second PC (PC2) was significant for tilTemra cells but not for pTemra cells. PC2 may represent the heterogeneity of tilTemra, presumably due to the differential contexts of each tumor microenvironment (TME). We further confirmed the observations with hierarchical clustering, which grouped tilTemra and pTemra into different clusters (figure 2B). The distance between each sample represented as ‘height’ was lower in pTemra and higher in tilTemra, demonstrating a higher heterogeneity among tilTemra cells.

Next, we analyzed differentially expressed genes between pTemra and tilTemra populations. Among the 15 299 genes analyzed, 1708 genes (11.16%) were differentially expressed (figure 2C). 910 genes (5.95%) were highly expressed in tilTemra, and 798 genes (5.22%) were highly expressed in pTemra. The expression of signature genes of differentiated cells, such as *TBX21*, *EOMES*, *PRF1*, *GZMB*, and *GZMH,*^4^ was upregulated in pTemra (online supplemental figure 2A). The expression of costimulatory molecules *ICOS* and *CD28*, which is downregulated in differentiated CD8^+^ T cells,^1 20^ was upregulated in tilTemra (online supplemental figure 2B). The expression of *B3GAT1* and *TOX*, markers for senescence or exhaustion,^3 21^ was upregulated in pTemra (online supplemental figure 2C), whereas the expression of *MYC* and *TCF7*, signatures for self-renewed stem cell-like properties,^21 22^ was upregulated in tilTemra (online supplemental figure 2D).

To obtain further insights into the functional differences between tilTemra and pTemra, we performed pathway analysis using the gene set enrichment analysis method. We obtained several sets of genes associated with differentiation, cytotoxicity, and proliferation from open resources (Gene Ontology and Broad Institute). Sets of genes associated with differentiation showed that the phenotype of tilTemra was closer to naive-like phenotype than that of pTemra (figure 2D top and online supplemental figure 2E). pTemra cells were enriched with sets of genes associated with cytotoxicity (figure 2D middle and online supplemental figure 2F), whereas tilTemra cells were enriched with sets of genes associated with proliferation (figure 2D bottom and online supplemental figure 2G). Together, these data indicate that tilTemra has a unique transcriptome distinct from terminally differentiated pTemra with less exhausted (*TBX21^lo^TOX^lo^*) naive (and stem)-like (*TCF7^hi^MYC^hi^*) gene expression profiles.

### tilTemra cells are phenotypically and functionally heterogeneous

**Figure 3:**
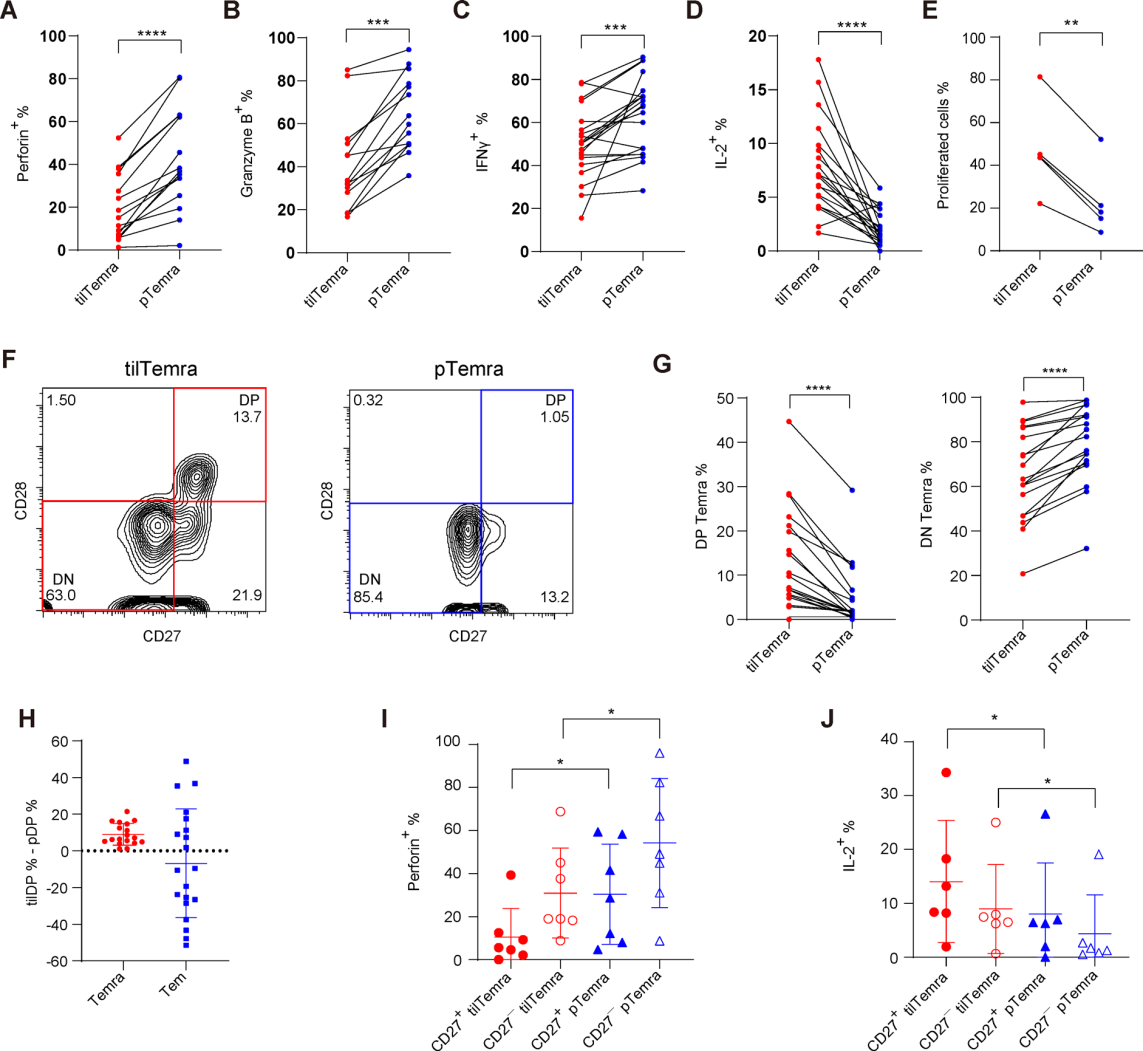
Comparison of functional phenotypes between tilTemra and pTemra. (A–D) TILs and PBMCs were restimulated for 4 hours and frequencies of (A) Perforin, (B) Granzyme B, (C) IFN-γ, and (D) IL-2 producing tilTemra or pTemra were assessed by flow cytometry. (n=14–20) (E) tilTemra and pTemra were purified and labeled with CTV, then stimulated with anti-CD3 (5 µg/mL), anti-CD28 (2 µg/mL), and IL-2 (10 ng/mL) for 7 days. Proliferated cells were assessed by flow cytometry. (n=5) (F) Representative flow cytometric data of CD27 and CD28 expression of tilTemra (left) and pTemra (right). (G) Proportions of DP (left) and DN (right) phenotypes of tilTemra or pTemra. (n=19) (H) The difference in proportion of DP phenotype was assessed by subtracting proportion of DP phenotype in PBMCs (pDP%) from proportion of DP phenotype in TILs (tilDP%). The difference (tilDP% − pDP%) was always above 0 (dotted line) in Temra but not in Tem. (n=19) (I, J) TILs and PBMCs were restimulated for 4 hours and frequencies of (I) Perforin and (J) IL-2 producing cells were assessed using flow cytometry. (n=6–7). (A–J) Statistical significance was performed with Wilcoxon matched-pairs signed rank test or (E) paired Student’s t-test. Values of *p<0.05, **p<0.01, ***p<0.001, ****p<0.0001 were considered significant. CTV, CellTrace Violet; DN, double-negative; DP, double-positive; IFN-γ, interferon γ; IL-2, interleukin 2; PBMCs, peripheral blood mononuclear cells; pTemra, peripheral blood Temra; Tem, effector memory T cells; TILs, tumor-infiltrating lymphocytes; tilTemra, tumor-infiltrating Temra.

Given that tilTemra cells were transcriptionally different from pTemra cells, we next examined the differences in their functional properties. In agreement with previous findings, pTemra cells showed a higher production of perforin, granzyme B, and IFN-γ, whereas tilTemra cells showed a higher production of IL-2 and a more potent proliferation capacity (figure 3A-E and online supplemental figure 3A). A similar pattern was observed between tilTem and pTem cells, where pTem cells showed a higher production of perforin and granzyme B (online supplemental figure 3B-E).

Based on the above functional data, tilTemra cells appeared to be functionally less differentiated than pTemra cells. The differentiation state of each subpopulation can be further defined by the expression of costimulatory receptors, CD27 and CD28.^1 11^ Although less differentiated cells display a double-positive (CD27^+^CD28^+^; DP) phenotype, more differentiated cells exhibit single-positive (CD27^+^CD28^−^ or CD27^−^CD28^+^; SP) or double-negative (CD27^−^CD28^−^; DN) phenotypes.^1^ tilTemra cells showed a higher proportion of DP phenotype than pTemra cells (figure 3F, G). Notably, the proportion of DP phenotype in tilTemra was always higher than that in pTemra, as shown by the equation ‘tilDP% – pDP%’. The value obtained was bigger than 0 for every patient (figure 3H), implying tumor-specific accumulation of DP Temra as a reliable, redundant phenomenon. In contrast to Temra, Tem showed no such consistency (figure 3H and online supplemental figure 3F), supporting the uniqueness of DP Temra cells.

To further confirm whether the distinct phenotypes of tilTemra cells were indeed tumor-specific rather than common nature of tissue-resident cells, we acquired normal tissues adjacent to tumor (NATs) and compared Temra cells from NATs (nTemra) with pTemra and tilTemra cells. nTemra cells showed lower proportion of DP phenotype than tilTemra cells (online supplemental figure 3G), as well as lower production of IL-2 (online supplemental figure 3H) and higher production of perforin (online supplemental figure 3I), further highlighting unique, tumor-specific phenotype and functionality of tilTemra cells.

We further examined whether the functional difference between tilTemra and pTemra subsets could be attributed to the differential proportion of DP cells in these two subsets. As expected, CD27^+^ Temra showed lower perforin production and higher IL-2 production than their CD27^−^ counterparts at the same origin (TILs or PBMCs) (figure 3I, J). However, even in the same CD27^+^ or CD27^−^ cells, the characteristics distinguishing tilTemra and pTemra populations were still preserved (figure 3I, J). Together with the transcriptional analysis shown in figure 2, these data suggest that tilTemra and pTemra are phenotypically and functionally different populations, with tilTemra (especially DP) exhibiting much less differentiated functional phenotype.

### CD8^+^ T cells show a bifurcated trajectory in Temra differentiation

**Figure 4:**
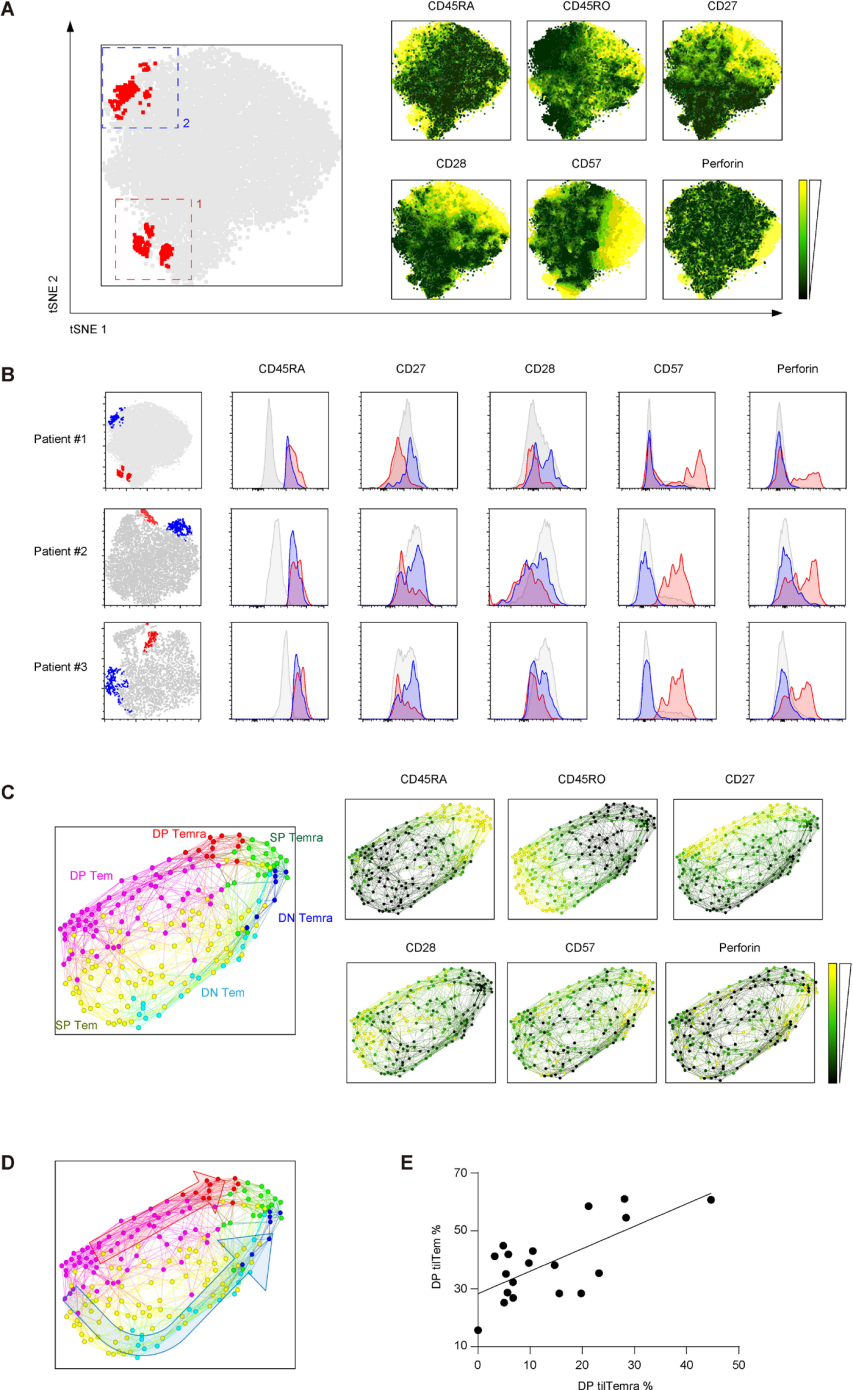
Differentiation trajectory of CD8^+^ TILs into Temra. (A, B) tSNE analysis of CD8^+^ TILs. TILs were gated to CD3^+^CD8^+^ cells prior to tSNE analysis. Following parameters were used for tSNE: CD27, CD28, CD45RA, CD45RO, CD8, CD3, CD5, CCR7, Perforin, and CD57. (A) Red dots (left panel) represent CD45RA^+^CD45RO^−^CCR7^−^ Temra. Two clustering (numbered 1 and 2) of tilTemra was observed. (B) Two clustering (colored red and blue) of tilTemra was observed in three different patients. Expression of indicated molecules were shown with the histogram. Gray histogram represents tilTem (C) Single cell trajectory analysis was performed with FLOW MAP using CD3^+^CD8^+^ TILs. Following clustering variables were used for FLOW MAP: CD27, CD28, CD45RA, CD45RO, CD8, CD3, CD5, and CCR7. Relative expression of CD27, CD28, CD45RA, and CD45RO of each node was assessed using Gephi software and colored accordingly (Pink: DP Tem, Yellow: SP Tem, Light blue: DN Tem, Red: DP Temra, Green: SP Temra, Dark blue: DN Temra). (D) A bifurcated differentiation trajectory illustrated by two arrows (red and blue). (E) Correlation between DP tilTem and DP tilTemra. (n=19) (A, C) Relative expressions of indicated molecules were represented by color scale from black (low) to yellow (high). CCR7, C-C chemokine receptor type 7; DN, double-negative; DP, double-positive; SP, single-positive; TILs, tumor-infiltrating lymphocytes; tilTemra, tumor-infiltrating Temra; tSNE, t-distributed stochastic neighbor embedding.

To further understand the differentiation and lineage relationship between DN and DP Temra, we applied the tSNE method to visualize the clustering of phenotypically similar cells from flow cytometry data.^23^ Notably, tilTemra subset was clustered into two distinct populations: (1) CD27^lo^CD28^lo^CD57^hi^Perforin^hi^ and (2) CD27^hi^CD28^hi^CD57^lo^Perforin^lo^ (figure 4A and online supplemental figure 4A). Such bifurcation of tilTemra subset was consistently observed in samples derived from different patients with NSCLC (figure 4B and online supplemental figure 4B), thus suggesting close and complex interplay of Temra differentiation with tumor.

We next sought to define the differentiation trajectory toward DP Temra by utilizing recently published FLOW-MAP.^18^ FLOW-MAP clusters phenotypically similar cells into ‘nodes’ and connects similar nodes with ‘edges’, generating a trajectory of nodes (figure 4C left). We labeled each node based on the expression of CD45RA, CD45RO, CD27, and CD28 (figure 4C right and online supplemental figure 4C). We observed a transition from the CD45RO^+^ Tem nodes (left) to CD45RA^+^ Temra nodes (right); however, the transition followed a bifurcated trajectory (figure 4D): (1) DP Tem → SP Tem → DN Tem → DN Temra (blue arrow) and (2) DP Tem → DP Temra (red arrow). SP Tem and SP Temra nodes were positioned between DP and DN nodes, bridging the transition from the DP to the DN phenotype. These findings strongly suggest that DP tilTemra cells are generated directly from DP tilTem cells surpassing the transitional state of SP or DN tilTem. In line with this hypothesis, we also observed a high correlation between the proportions of DP tilTem and DP tilTemra (figure 4E).

### Strength of TCR engagement determines the fate of Temra differentiation

**Figure 5:**
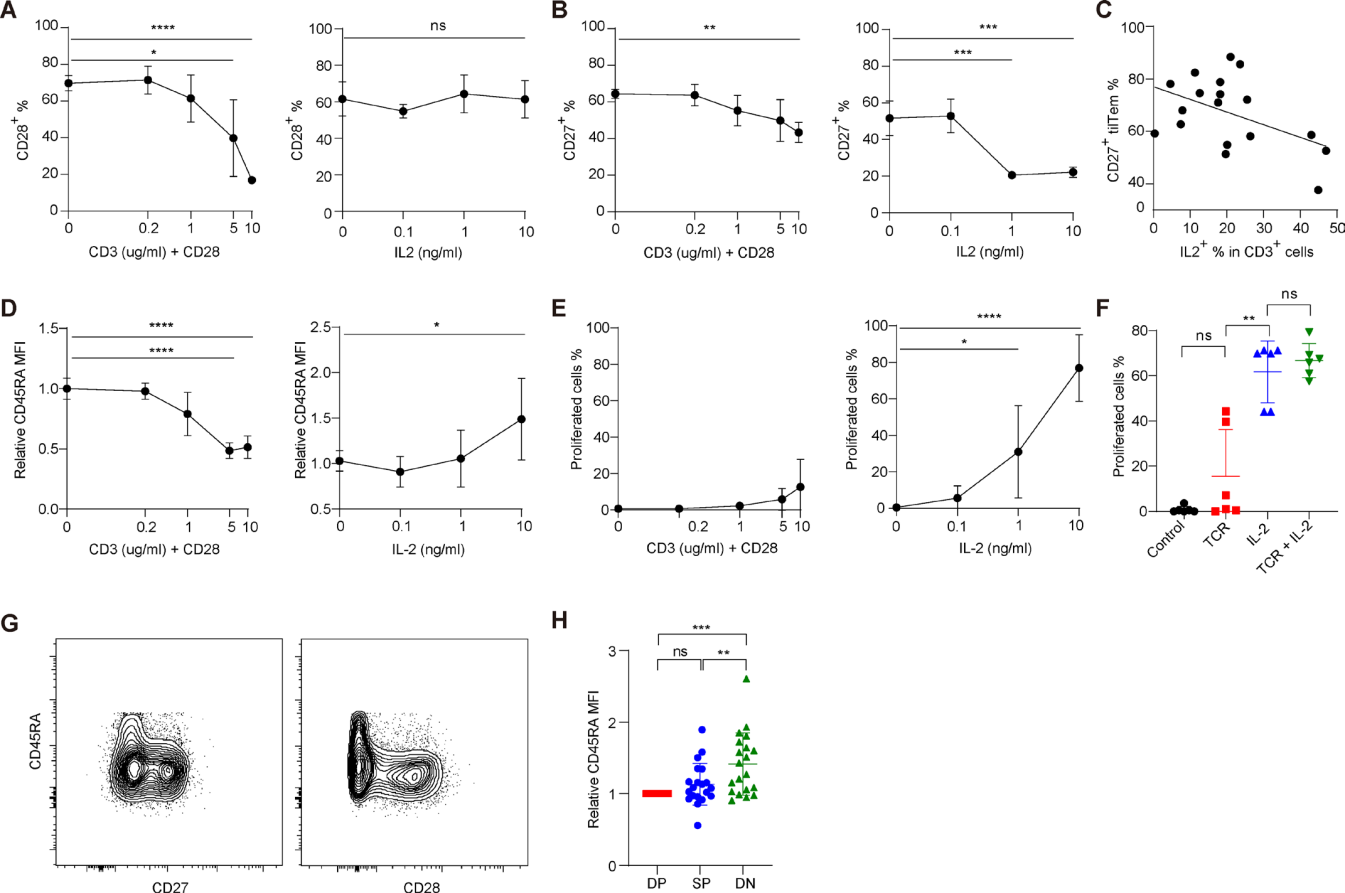
Regulation of the expression of CD27, CD28, and CD45RA in Temra differentiation. (A, B) tilTem was purified and stimulated with anti-CD3 (0~10 µg/mL) and anti-CD28 (0~4 µg/mL) (left) or with IL-2 (0~10 ng/mL) (right) for 7 days. (n=3–5) Expressions of (A) CD28 and (B) CD27 were assessed by flow cytometry. (C) TILs were restimulated for 4 hours and frequency of IL-2 producing CD3^+^ cells were analyzed by flow cytometry. Proportion of IL-2^+^ in CD3^+^ cells were plotted along with proportion of CD27^+^ in tilTem. (n=18) (D, E) tilTem was labeled with CTV, then stimulated with indicated stimulations. (n=4–5) After 7 days, (D) CD45RA expression and (E) percentage of proliferated cells were assessed by flow cytometry. Relative CD45RA MFIs were calculated by dividing by the average CD45RA MFI of unstimulated cells. (F) tilTem was labeled with CTV, then stimulated with TCR (anti-CD3 (5 µg/mL) and anti-CD28 (2 µg/mL)), IL-2 (10 ng/mL), or both. Proliferated cells were assessed after 7 days. (n=5) (G) Representative flow cytometric data indicating re-expression of CD45RA in tilTem. CD45RA^lo^CCR7^−^ tilTem was gated from TILs and expression of CD45RA was assessed with either CD27 (left) or CD28 (right). (H) CD45RA expression of DP, SP, and DN tilTem. Relative CD45RA MFIs were calculated by dividing by the MFI value of DP cells from the same tumor. (n=20) (A–H) Data show mean±SD. Statistical significance was performed with (A–F) unpaired Student’s t-test or (H) Wilcoxon matched-pairs signed rank test. Values of *p<0.05, **p<0.01, ***p<0.001, ****p<0.0001 were considered significant. CTV, CellTrace Violet; DN, double-negative; DP, double-positive; IL-2, interleukin 2; ns, not significant; SP, single-positive; TCR, T cell receptor; MFI, mean fluorescence intensity.

Next, we investigated the mechanism underlying divergent differentiation into either DN or DP tilTemra. The key requirement for this bifurcation is either timely upregulation or downregulation of the expression of CD27 and CD28 while re-expressing CD45RA, a hallmark of Temra, during tumor progression. We, thus, examined a role of antigenic stimulation via TCR engagement and of cytokines especially those produced by various cell types in TME. For this, we FACS-purified tilTem and cultured them with varying doses of anti-CD3/CD28 or with various cytokines. Unlike Tn (online supplemental figure 5A), tilTem significantly downregulated CD28 on anti-CD3/CD28 stimulation (figure 5A left); however, CD27 expression in tilTem was largely unchanged although moderately decreased only at a high concentration of anti-CD3 (figure 5B left).

Among various cytokines examined under in vitro conditions, IL-2 induced a substantial down-regulation of CD27 (figure 5B right and online supplemental figure 5B) but not of CD28 expression (figure 5A right and online supplemental figure 5C), which was in sharp contrast to TCR ligation. In line with this finding, the proportion of CD27-expressing (CD27^+^) tilTem cells showed a significant inverse correlation with the proportion of IL-2-producing (IL-2^+^) total CD3^+^ T cells (figure 5C). Since IL-2 is mainly produced by activated CD3^+^ T cells (both CD4^+^ and CD8^+^) after anti-CD3/CD28 stimulation (online supplemental figure 5D),^24^ it seems conceivable that TCR stimulation modulates CD27 and CD28 expression either directly or indirectly (via IL-2). Hence, these results indicate that strong TCR engagement promoted the downregulation of CD27 and CD28 and accordingly promoted the differentiation of DN Temra cells but inhibited that of DP Temra cells.

Considering the role of TCR engagement in the regulation of CD27 and CD28 expression, we next examined its influence on CD45RA expression, which should be upregulated during Temra differentiation. The expression of CD45RA, despite being very low in tilTem cells, was further downregulated after anti-CD3/CD28 stimulation (figure 5D left), which was consistent with a previous report.^6^ Similar downregulation of CD45RA was observed even in Temra cells (online supplemental figure 5E), indicating that strong antigenic stimulation should be avoided for upregulating CD45RA during the transition of tilTem to tilTemra. Notably, IL-2 treatment, unlike CD27, induced the upregulation of CD45RA expression, which was in marked contrast to TCR engagement. However, this required relatively high doses of IL-2 (figure 5D right), suggesting a potential role of IL-2 in regulating the differentiation of Temra cells.

Since Terma cells have a short telomere length (figure 1C),^9^ presumably due to prolonged, recurrent proliferation, we investigated whether tilTem cells could indeed proliferate consistently in response to TCR stimulation. Notably, tilTem cells proliferated poorly on anti-CD3/CD28 engagement (figure 5E left and online supplemental figure 5F); however, they showed vigorous proliferation on IL-2 exposure (figure 5E right and online supplemental figure 5G). Proliferation was prominent with IL-2 alone and was barely affected even with additional TCR engagement (figure 5F). These data, along with previous reports showing poor responsiveness of Tem cells to TCR stimulation,^25–27^ indicate that TCR signaling may be dispensable or timely suppressed (via an active mechanism such as TCR desensitization) during the differentiation of tilTemra cells.

Supporting this observation, the re-expression of CD45RA in tilTem cells was much more pronounced in CD27^−^ and CD28^−^ cells—that is, the cells lacking costimulatory molecules required for productive TCR signaling—but not in CD27^+^ and CD28^+^ cells (figure 5G). Similarly, expression levels of CD45RA significantly increased as tilTem cells differentiated from DP to SP to DN states (figure 5H). Similar results were also observed in pTem cells (online supplemental figure 5H and I). Together, these data strongly suggest that only a negligible or a low level of TCR signaling (in conjunction with the loss of costimulatory molecules) promotes the re-expression of CD45RA in Tem (or Teff) cells and their transition into either DN or DP Temra cells.

### Proportion of DP Temra cells is inversely correlated with the number of CD8^+^ TILs and TMB

The main implication from the above results was that strong, recurrent antigenic stimulation via TCR ligation with tumor antigens would negatively influence Temra (especially DP Temra) differentiation. This finding is in agreement with previous reports demonstrating that CD27^−^CD28^−^ DN Temra population is more prevalent in chronically infected viral antigen-specific CD8^+^ T cells.^2 28 29^ Hence, the important question of how tumor antigen-specific CD8^+^ Tem (or Teff) cells can differentiate into DP Temra rather than DN Temra arises. Given that high doses of anti-CD3/CD28 treatment downregulated the expression of CD27 and CD28 (figure 5A, B), we postulated that lowering TCR stimulation—below a level that does not trigger DP to DN transition—would be crucial to maintain constantly high levels of these costimulatory molecules throughout Temra differentiation during tumor progression.

**Figure 6:**
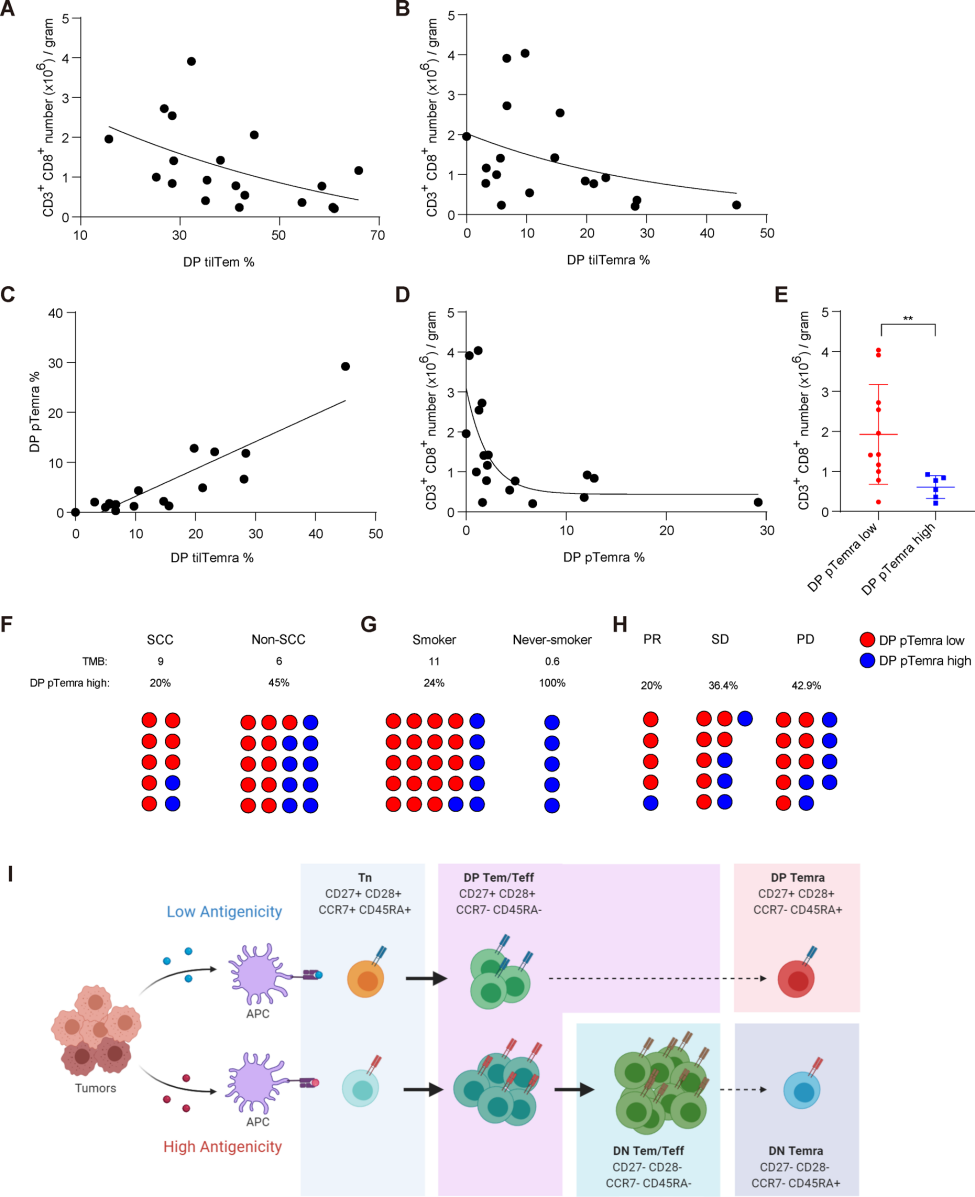
Correlation between the proportion of DP Temra and the number of CD8^+^ TILs and TMB. (A–D) Correlations were assessed between (A) number of CD8^+^ TILs and proportion of DP tilTem, (B) number of CD8^+^ TILs and proportion of DP tilTemra, (C) proportion of DP pTemra and proportion of DP tilTemra, and (D) number of CD8^+^ TILs and proportion of DP pTemra. (n=19) (E) Number of CD8^+^ TILs in patients with low (<6%) or high (>6%) proportion of DP pTemra. (F, G) Blood of NSCLC patients were harvested and proportion of DP pTemra was analyzed by flow cytometry, then assessed how many DP pTemra high patients were in (F) SCC (9 coding somatic mutations per MB; TMB: 9), non-SCC (TMB: 6), (G) Smoker (TMB:11), and never-smoker (TMB: 0.6). (n=30), (H) or in patients with PR, SD, PD in response to immune checkpoint inhibitors. (I) Schematic model describing how tumor antigens shape the differentiation trajectory of CD8^+^ TILs (Created with BioRender.com). (E) Data show mean±SD. Statistical significance was performed with unpaired Student’s t-test. Values of *p<0.05, **p<0.01, ***p<0.001, ****p<0.0001 were considered significant. DP, double-positive; NSCLC, non-small cell lung cancer; PD, progressive disease; PR, partial response; pTemra, peripheral blood Temra; SCC, squamous cell carcinoma; SD, steady disease; tilTemra, tumor infiltrating Temra; tilTem, tumor infiltrating Tem; TMB, tumor mutation burden.

Tumor antigens such as tumor-associated antigens (TAAs) and neoantigens (associated with TMB) vary in their stimulatory capacities for antigen-specific naive CD8^+^ T cells^30^ and CD8^+^ TILs,^31 32^ as different levels of TCR engagement could induce Tn cell expansion to variable extents (online supplemental figure 6A). Hence, we speculated that TAAs vs neo-antigens may have different fate choices, considering DN and DP Temra differentiation. Notably, the number of CD8^+^ TILs derived from patients with NSCLC was inversely correlated with the proportion of DP tilTem (figure 6A) as well as DP tilTemra (figure 6B). Therefore, these data strongly support our hypothesis that low immunogenic stimulation with tumor antigens (eg, TAAs) leads to sustained expression of CD27 and CD28 in tumor-specific CD8^+^ T cells and promotes their differentiation into DP Temra over DN Temra.

Furthermore, we observed a strong correlation in the proportions of DP pTemra and DP tilTemra (figure 6C), suggesting that these two subsets share some degree of specificity with a given tumor antigen and circulate between the tumor site and peripheral blood. Additionally, the proportion of DP pTemra was inversely correlated with the number of CD8^+^ TILs (figure 6D). Accordingly, patients with a relatively low proportion of DP pTemra (<6%) demonstrated twice more CD8^+^ TILs than patients with a high proportion of DP pTemra (>6%; figure 6E), allowing us to predict the number of CD8^+^ TILs simply by analyzing DP Temra cells in the blood of patients with NSCLC.

To gain more insight into possible relationship between DP tilTemra and DP pTemra, we examined TCR α/β usages within CD8^+^ T cell subsets (CD27^+^/CD27^−^ Tem and CD27^+^/CD27^−^ Temra) in PBMCs and TILs from six patients with NSCLC. Among these subsets, CD27^+^ pTemra and CD27^+^ tilTemra cells appeared to share the most similar degree of TCR Vα7.2, Vβ8, Vβ12 chain usages (red boxes; online supplemental figure 6B-D). Notably, CD27^+^ tilTemra and CD27^−^ tilTemra cells exhibited distinctly different levels of TCR Vα7.2, Vβ8, Vβ12 chain usages (blue box; online supplemental figure 6B-D), implying little relationship between these subsets. To further confirm the above notion, we analyzed additional TCR α/β usages (Va2, Vα7.2, Vα12.1, Vβ3, Vβ5b, Vβ8, Vβ12, and Vβ13.1) of CD27^+^/CD27^−^ Tem and CD27^+^/CD27^−^ Temra cells from PBMCs or TILs, then calculated their similarity of the 8 TCR α/β usages between each sample using sample distance matrix (DESeq2). CD27^+^ tilTemra and CD27^+^ pTemra cells were among the closest samples (red line; online supplemental figure 6E), while CD27^−^ tilTemra cells were always distant from them (blue line).

To further confirm the afore-mentioned strong inverse correlation between the proportion of DP pTemra and the number of CD8^+^ TILs along with the degree of tumor immunogenicity (TMB), we newly collected PBMCs from 30 patients with a terminal stage of NSCLC (online supplemental table 2) and divided them into two groups according to the proportion of DP pTemra. The group with lower proportion of DP pTemra cells (DP pTemra% <6%) showed increased Ki-67^+^ (online supplemental figure 6F) and PD-1^+^ (online supplemental figure 6G) CD8^+^ T cells compared with the group with a higher proportion of DP pTemra cells (DP pTemra% >6%), suggesting a more robust immune response.

Next, to examine whether the observed difference between these two groups indeed correlates with the degree of tumor immunogenicity, we sorted the clinical information of the patients based on a TMB score reported previously.^33–35^ Squamous cell carcinoma (SCC) was reported to have a higher TMB than non-SCC.^33 34^ Accordingly, 20% of patients with SCC (TMB: 9) and 45% of patients with non-SCC (TMB: 6) were classified into the group with a high proportion of DP pTemra cells (figure 6F and online supplemental figure 6H). Likewise, smoking history is a critical factor for NSCLC TMB.^35^ While only 24% of smokers (TMB: 11) were classified into the group with a high proportion of DP pTemra cells, all never-smokers (individuals who never smoked; TMB: 0.6) were classified into the said group (figure 6G and online supplemental figure 6I).

Number of CD8^+^ TILs and TMB are two of the most widely used and clinically validated biomarkers for cancer immunotherapy.^36–39^ Since patients with high CD8^+^ TILs or TMB correlate well with better response to immune checkpoint inhibitors (ICIs; eg, anti-programmed cell death protein 1 (PD1) or anti-programmed death ligand 1 (PD-L1)),^36–39^ we speculated that patients with low proportion of DP pTemra cells are more likely to show good response to ICIs. To test this hypothesis, after the blood analysis (figure 6F–G), patients were treated with ICIs (anti-PD1/PD-L1; online supplemental table 2). We monitored the patients’ responses to the ICIs, then compared the clinical outcome with the proportion of DP pTemra cells obtained before treatment. While only 20% of the patients with partial response (PR) were in the group with a high proportion of DP pTemra cells, 36.4% and 42.9% of the patients with steady disease (SD) and progressive disease (PD) were with a high proportion of DP pTemra cells, respectively (figure 6H).

Collectively, these data further strengthen our notion that the proportion of DP pTemra is inversely correlated with the immunogenic potential of tumor antigens (ie, TMB) and the number of CD8^+^ TILs, providing valuable clinical implication for predicting response outcome of patients receiving cancer immunotherapy.^36–39^

## Discussion

Phenotypic analyses of tumor-infiltrating CD8^+^ T cells have been widely performed.^1–3^ Since most CD8^+^ TILs have CCR7^−^CD45RA^−^ Teff/Tem phenotypes,^13–15^ Temra cell population has often been neglected, and its physiological relevance remains elusive. In this study, we focused on tilTemra derived from patients with NSCLC and characterized previously undefined unique differentiation patterns associated with the degree of immunogenicity of developing tumors. The tilTemra cells showed a distinct phenotype from pTemra with higher proportion of CD27^+^CD28^+^ DP and conversely lower proportion of CD27^−^CD28^−^ DN phenotype, along with transcriptomic and functional differences. Single-cell trajectory analysis revealed that tumor-infiltrating CD8^+^ T cells follow a divergent differentiation path: (1) DP Tem → SP Tem → DN Tem → DN Temra or (2) DP Tem → DP Temra. Importantly, in this bifurcated trajectory, the strength of TCR engagement with tumor-derived antigens was a key limiting step in determining the direction of differentiation. As such, relatively weak TCR stimulation was crucial for DP Tem → DP Temra transition. Consequently, the proportion of DP Temra cells inversely correlated with the number of CD8^+^ TILs and TMB. Hence, based on these data, we demonstrate DP Temra cells—analyzed in the tumor specimens and even in the blood samples of the patients—as a novel means for predicting the immunogenicity of tumor antigens and accordingly, CD8^+^ TIL counts, a reliable biomarker for successful cancer immunotherapy.

Precisely, how could a tumor-derived antigen shape such bifurcation in the differentiation of CD8^+^ TILs into Temra? Our model for the divergent differentiation trajectory into either DP or DN Temra is illustrated in figure 6I. Tumor antigens with high immunogenicity would induce robust expansion of antigen-specific naive CD8^+^ T cells in tumor-draining lymph nodes. The Tn cells express high levels of CD27 and CD28 and further upregulate the expression of these costimulatory molecules on activation. Thus, the recently activated, early-stage Teff cells would exhibit the CD27^+^CD28^+^ DP phenotype. However, in the later stage Teff, persistent recurrent exposure of the immunogenic tumor antigens leads to a gradual loss of the expression of CD27 and CD28, converting DP cells to DN cells. From this point, DN Teff cells become reluctant to continuous TCR engagement (‘desensitized’ to the cognate tumor antigens) but are still responsive to IL-2 available in the TME, promoting re-expression of CD45RA for the differentiation of DN Temra cells. In sharp contrast to the fate of DN Temra cells, tumor antigens with low immunogenicity induce weak activation/proliferation of Tn cells, generating a relatively small number of DP Teff cells. However, prolonged low immunogenic TCR engagement would be insufficient to trigger CD27 and CD28 downregulation, allowing these Teff cells to sustain DP phenotype and eventually to become DP Temra cells re-expressing CD45RA.

Since the majority of Temra cells found in the peripheral blood of healthy individuals exhibit a DN and rarely DP phenotype,^1 11^ DP Temra cells observed in patients with cancer were intriguing and raised the question of how this particular subset is generated and whether its appearance is linked to tumor. In chronic viral infections, repeated exposure to the same viral antigens induces the production of Temra cells.^2 28 29^ For example, human cytomegalovirus (HCMV) induces a series of recurrent activation of viral antigen-specific CD8^+^ T cells and generates Temra cells,^2 29^ with HCMV-specific Temra cells exhibiting the DN phenotype.^40 41^ On analyzing these previous observations with the afore-mentioned model, it was observed that viral peptides were typically highly immunogenic foreign antigens that resulted in potent T cell activation via strong TCR engagement. Therefore, viral antigen-specific CD8^+^ Teff (or Tem) cells are likely to differentiate into DN Temra cells. Likewise, most clinical cases with infections and diseases involving strong CD8^+^ T cell responses against an immunogenic foreign antigen would be associated with preferential skewing toward DN over DP Temra cells, which explains why DP Temra subset has been barely recognized in these conditions.

Given the fact that Temra is the most terminally differentiated subset after chronic antigenic stimulation, the observed negative influence of the strength of TCR engagement on DP Temra generation was odd at first glance. Hence, we postulated that tumor antigens with relatively low immunogenicity are related to the shaping of the fate of DP Temra cells. The majority of patients with cancer demonstrate the expression of TAAs, which are known as self-antigens and are over-expressed in many tumors.^30^ However, TAAs typically exhibit low immunogenicity and hence induce weak T cell activation.^30^ In light of this view, previous studies demonstrated that CD8^+^ T cells specific to TAAs are CD27^+^ cells.^42^ In contrast to TAAs, neoantigens, which are generated as a result of genetic mutations in developing tumors, are often highly immunogenic non-self-antigens.^30^ Hence, in agreement with our model, neo-antigen-specific CD8^+^ T cells were shown to have all transitory subpopulations required for differentiating into DN Temra cells, that is, CD27 and CD28 DP, SP, and DN phenotypes.^42 43^ Moreover, given that the neoantigen diversity and specificity is highly correlated with TMB,^44^ our results stating that lung cancer patients with high TMB have a higher proportion of DN Temra cells but a lower proportion of DP Temra cells than those with low TMB provide strong support for the close relationship between the degree of TMB and the generation of highly immunogenic neoantigens and CD8^+^ TILs. However, the exact TMB analysis (eg, whole exome sequencing) will be needed to properly validate the relationship between the proportion of DP Temra and TMB.

The significant inverse correlation between the proportion of DP Temra cells (analyzed in the blood of patients with NSCLC) and the number of CD8^+^ TILs and TMB is of particular importance, as monitoring PBMCs is a routine procedure in the clinics with easier and faster readouts than those of surgical procedures. As CD8^+^ TILs and TMB are both well-established prognostic and predictive markers for cancer immunotherapy using ICIs, such as anti-CTLA4, anti-PD-1, and anti-PD-L1 monoclonal antibodies,^36–39^ whether patients with a relatively low proportion of DP Temra in peripheral blood indeed respond better to checkpoint blockade therapies will be interesting to explore. In this regard, we demonstrated that patients with better response to anti-PD-1/PD-L1 were mostly associated with low proportion of DP Temra cells, thus providing a potential use of DP pTemra as a predictive biomarker to diagnose patients with various types of cancer.

Although tilTemra cells reside in a highly immunosuppressive condition of TME, DP tilTemra cells were not functionally impaired. Further, tilTemra cells demonstrated less exhausted and self-renewed stem-like gene expression profiles (eg, *TOX^lo^, MYC^hi^,* and *TCF7^hi^*) compared with pTemra cells in the transcriptomic analysis performed by bulk RNA-seq. In line with this, DP tilTemra did not express CD57, a senescence maker indicating the ability of proliferation and differentiation plasticity.^3^ Since tilTemra express higher levels of *TCF7* transcript, a master signature for stem cell memory T cells (Tscm) that are primary target for ICIs,^45 46^ than pTemra, one may expect that it would be correlated with a better response to ICIs. Also, whether DP tilTemra cells that are reactive to tumor antigens can be used as a starting population for in vitro expansion in adoptive T cell therapy is intriguing and needs to be addressed in the future.

In summary, tumor-specific CD8^+^ T cells in patients with NSCLC demonstrate divergent differentiation fates toward Temra subset, largely with two distinct phenotypes based on the expression of CD27 and CD28 (CD27^−^CD28^−^ DN and CD27^+^CD28^+^ DP). Such bifurcation depends on the antigenic strength and immunogenic spectrum of growing tumors, resulting in a strong inverse correlation between DP Temra cells and the number of CD8^+^ TILs and TMB. Accordingly, proportion of DP Temra cells inversely correlates with the responsiveness to checkpoint blockade therapies. Additional investigation of other types of cancers, such as melanoma, will be important to further validate this phenomenon.

## Data Availability

Data are available in a public, open access repository. Data are available on reasonable request. All data relevant to the study are included in the article or uploaded as online supplemental information. The GEO accession number is GSE184053.
