# Supplementary material for: CD8+ TILs in NSCLC differentiate into TEMRA via a bifurcated trajectory: deciphering immunogenicity of tumor antigens
Source: J Immunother Cancer. 2021 Sep 30;9(9):e002709. doi: 10.1136/jitc-2021-002709 (PMC8487216; doi:10.1136/jitc-2021-002709)
Supplement: Supplementary data [file jitc-2021-002709supp001.pdf]

SUPPLEMENTARY MATERIALS

SUPPLEMENTARY DATA

Supplementary Figure 1

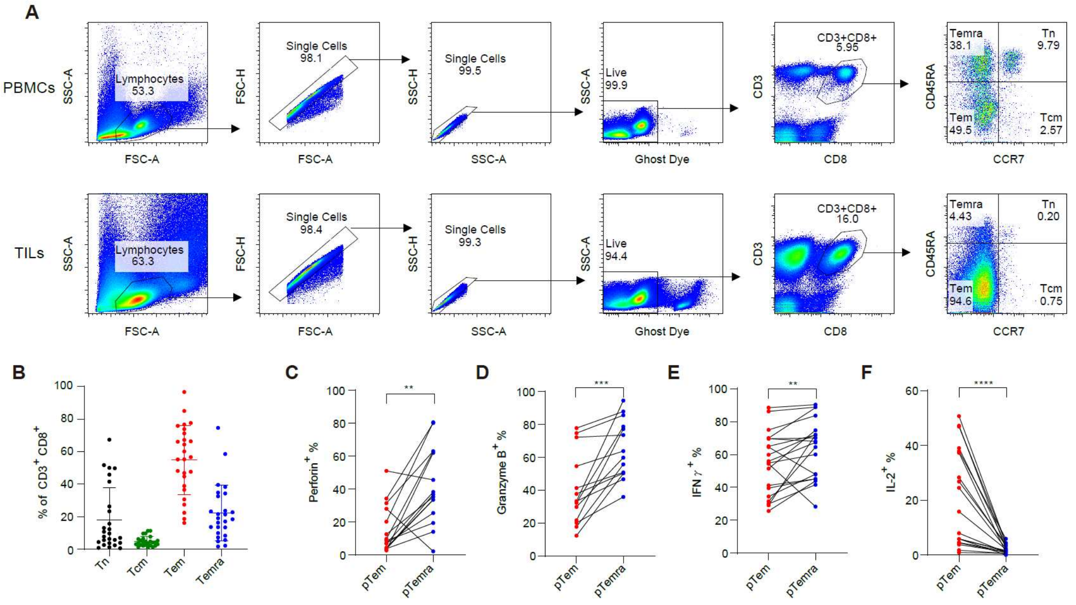

Figure. S1 Gating strategy and characteristics of peripheral CD8$^+$ T cell subpopulations. (A) Gating strategy to distinguish CD8$^+$ T cell subpopulations from PBMCs and TILs. (B) Proportions of CD8$^+$ T cell subpopulations in CD8$^+$ T cells from PBMCs. (n=26) (C-F) PBMCs were restimulated for 4 hours and the frequencies of (C) Perforin, (D) Granzyme B, (E) IFN-γ, and (F) IL-2 producing pTem or pTemra were assessed by flow cytometry (n=14-20). (C-F) Statistical significance was performed with Wilcoxon matched-pairs signed rank test.

Supplementary Figure 2

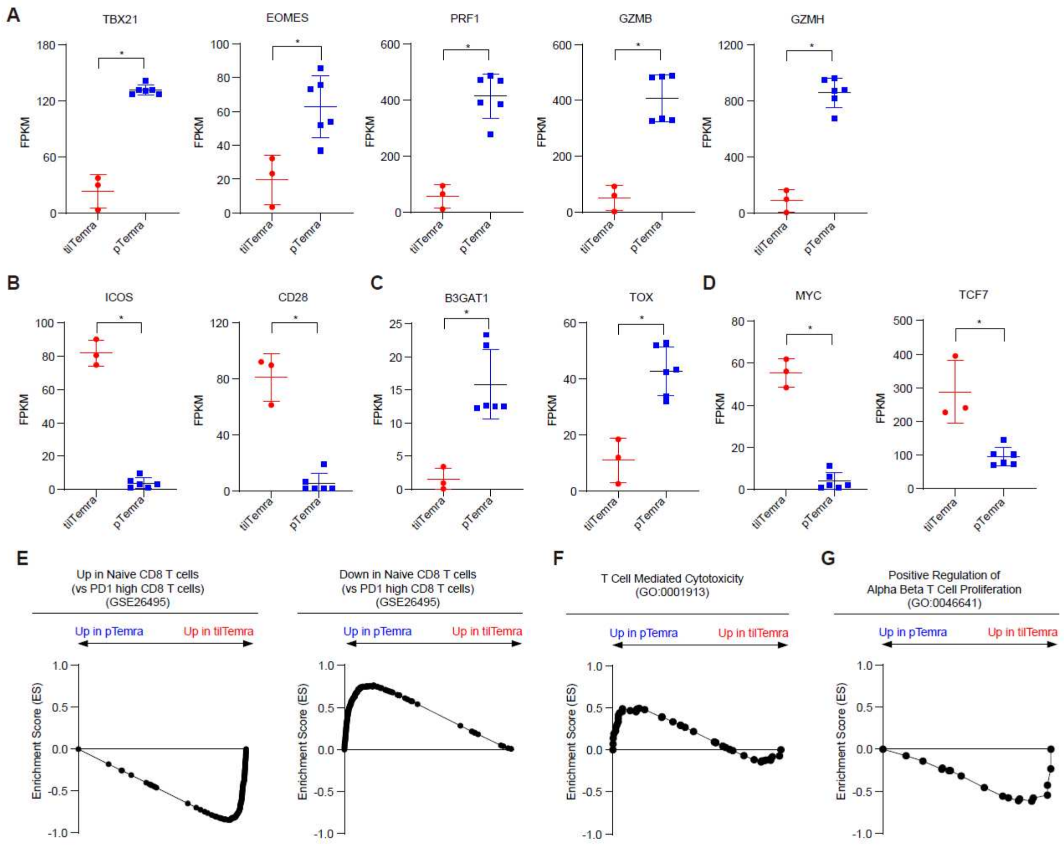

Figure. S2 Transcriptome differences between tilTemra and pTemra. (A-D) FPKM values from RNA seq data of genes related to (A) differentiation (TBX21, EOMES, PRF1, GZMB, GZMH), (B) co-stimulatory molecules (ICOS, CD28), (C) senescence or exhaustion (B3GAT1, TOX), and (D) proliferation or memory (MYC, TCF7). (E-G) GSEA results using gene sets related to (E) exhaustion, (F) cytotoxicity, and (G) proliferation. (A-D) Data show mean ± SD. Statistical significance was performed with Mann-Whitney test.

Lee S-W, *et al. J Immunother Cancer* 2021; 9:e002709. doi: 10.1136/jitc-2021-002709

Supplementary Figure 3

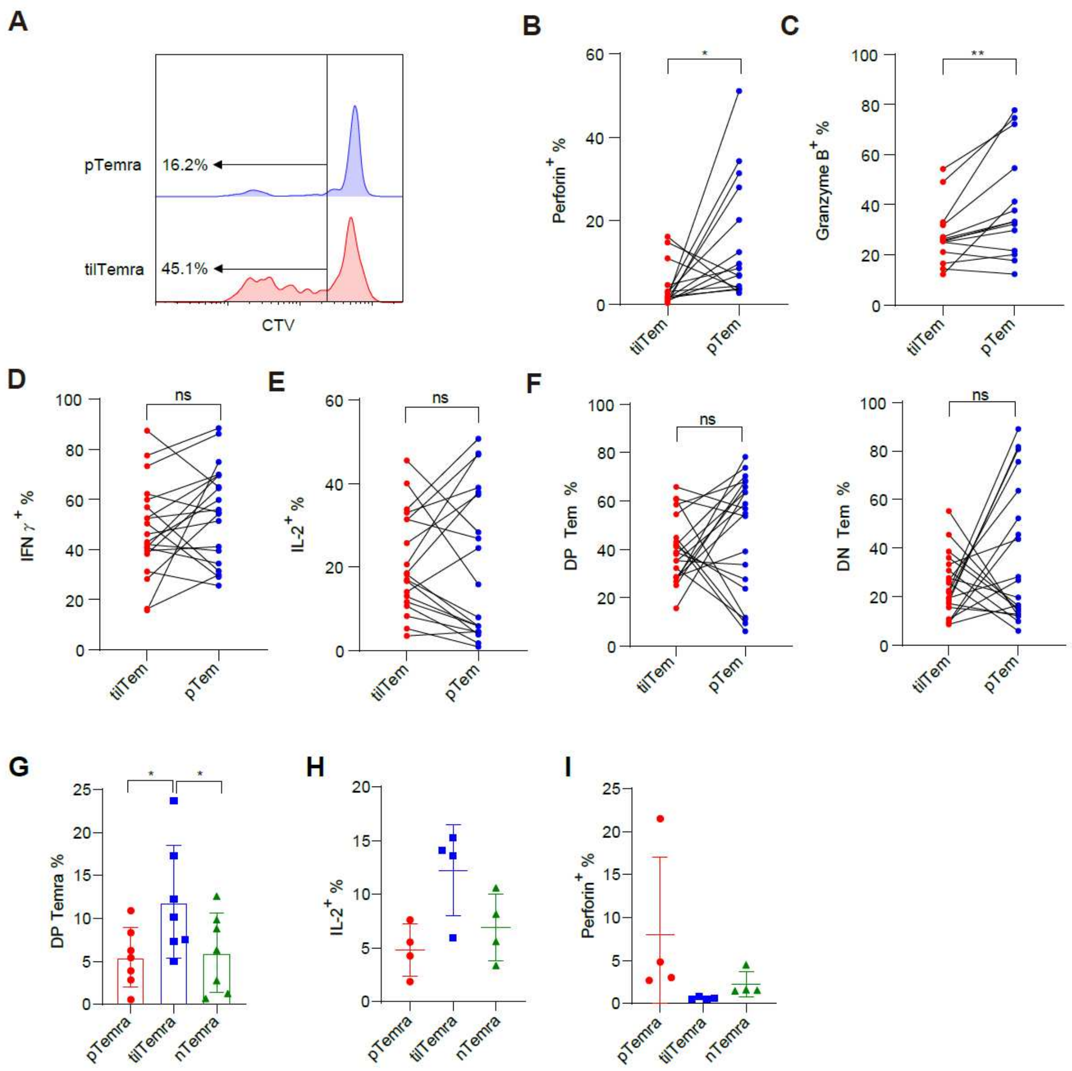

Figure. S3 Functional differences between tilTem and pTem. (A) Representative histogram of CTV dilution of cells stimulated with anti-CD3 (5 μg/ml), anti-CD28 (2 μg/ml), and IL-2 (10 ng/ml) for 7 days. (B-E) TILs and PBMCs were restimulated for 4 hours and the frequencies of (B) Perforin, (C) Granzyme B, (D) IFN-γ, and (E) IL-2 producing tilTem or pTem were assessed by flow cytometry. (n=14-20) (F) Proportions of DP (left) and DN (right) phenotypes from tilTem and pTem. (n=19) (G-H) Temra cells from PBMCs (pTemra), TILs (tilTemra), and NATs (nTemra) were analyzed for (G) proportion of DP, (H) IL-2 production,

and (I) perforin production. Statistical significance was performed with (B-F) Wilcoxon matched-pairs signed rank test or (G) Mann-Whitney test.

Lee S-W, *et al. J Immunother Cancer* 2021; 9:e002709. doi: 10.1136/jitc-2021-002709

Supplementary Figure 4

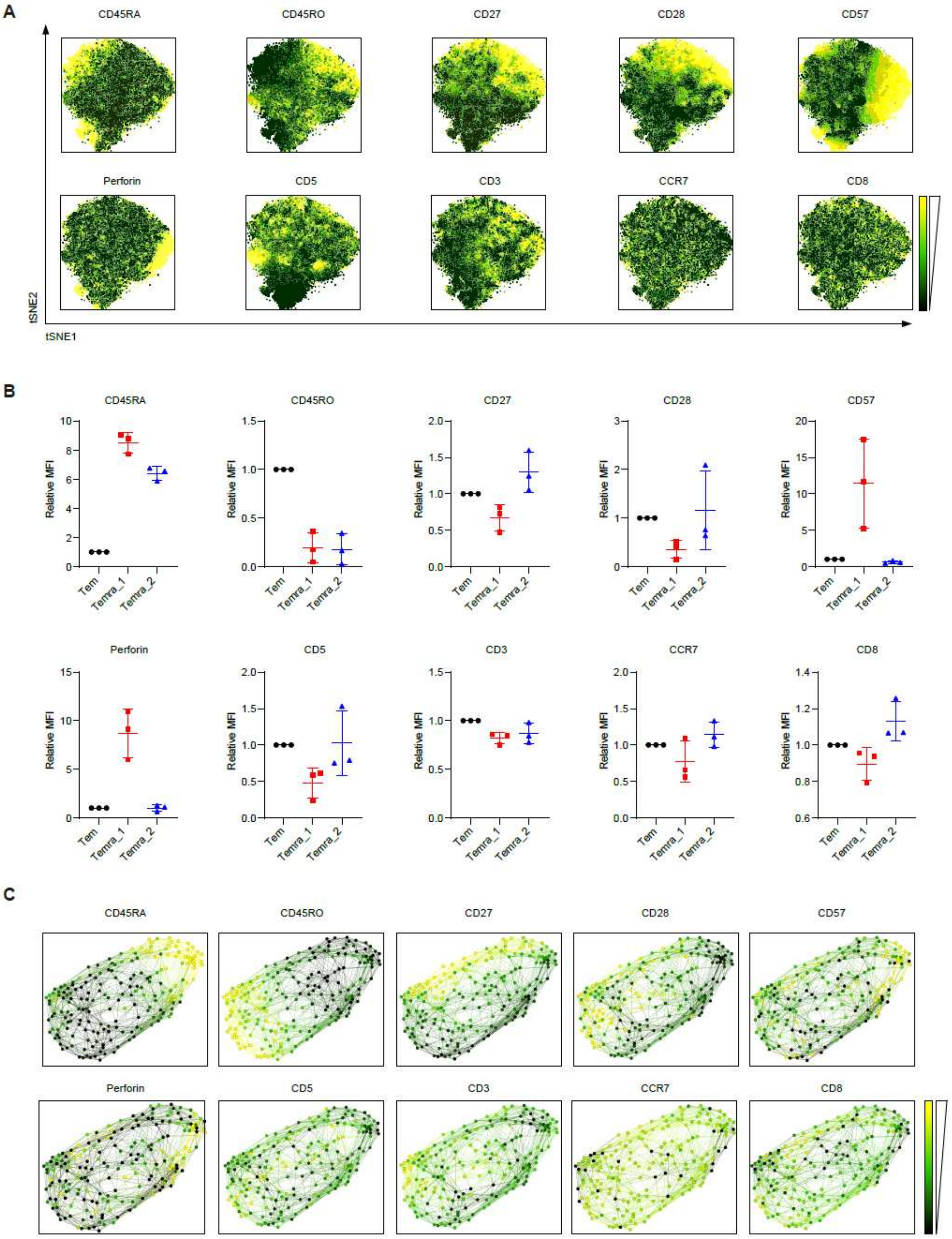

Figure. S4 Expressions of key molecules of CD8$^+$ TILs in tSNE and FLOW MAP. (A) Expression of indicated molecules in tSNE analysis of CD8$^+$ TILs. (B) Relative expression of

indicated molecules of tilTem and two clustering of tilTemra. Relative MFIs were calculated by dividing by MFI value of Tem cells from the same tumor. (C) Relative expression of indicated molecules in FLOW MAP result. (A and C) Relative expressions of indicated molecules were represented by color scale from black (low) to yellow (high).

Lee S-W, *et al. J Immunother Cancer* 2021; 9:e002709. doi: 10.1136/jitc-2021-002709

Supplementary Figure 5

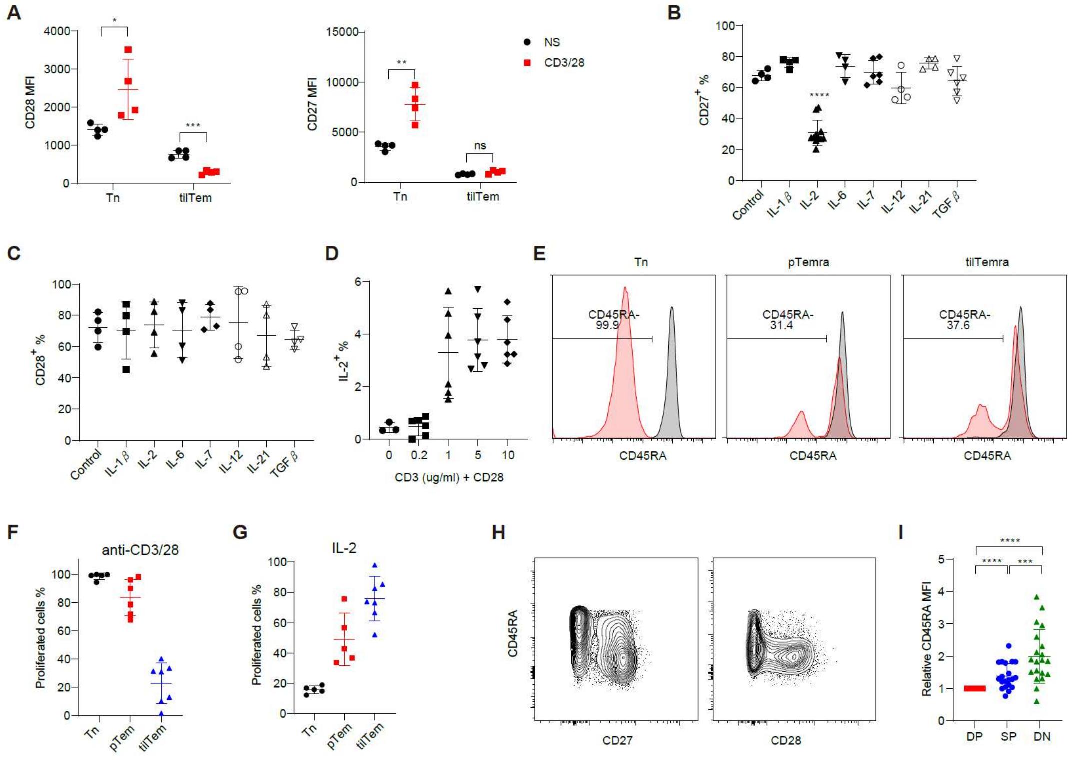

Figure. S5 Regulation expressions of CD27, CD28, and CD45RA. (A) Tn was purified and cultured with no stimulation (NS) or with anti-CD3 (5 μg/ml) and anti-CD28 (2 μg/ml) for 7 days. Expression of CD28 (left) and CD27 (right) were assessed with flow cytometry. (n=4) (B and C) tilTem was purified and cultured with indicated cytokines (20ng/ml xeach) for 7 days. Expression of (B) CD27 and (C) CD28 were assessed with flow cytometry. (n=4) (D) TILs were restimulated with indicated dose of anti-CD3 and anti-CD28 for 5 hours. GolgiPlug was added after the first hour. IL-2 producing tilTem was assessed with flow cytometry. (n=3) (E) Tn, pTemra, and tilTemra were purified and stimulated with no stimulation (Grey) or with anti-CD3 (5 μg/ml) and anti-CD28 (2 μg/ml) (Red) for 7 days. Expression of CD45RA was assessed with flow cytometry. (F and G) Tn, pTem, and tilTem were purified and labelled with CTV, then stimulated with (F) anti-CD3 (5 μg/ml) and anti-CD28 (2 μg/ml) or with (G) IL-2 (10ng/ml) for 7 days. Proliferated cells were assessed by flow cytometry. (n=3) (H)

Representative flow cytometric data indicating re-expression of CD45RA in pTem. CD45RA^{lo}CCR7^{-} pTem was gated from PBMCs and expression of CD45RA was assessed with either CD27 (left) or CD28 (right). (I) Relative CD45RA expression of DP, SP, and DN pTem. Relative CD45RA MFIs were calculated by dividing by the MFI value of DP cells from the same PBMCs (n=20) (A-I) Data show mean ± SD. Statistical significance was performed with (A-B) unpaired Student’s t-test or (I) Wilcoxon matched-pairs signed rank test.

Lee S-W, *et al. J Immunother Cancer* 2021; 9:e002709. doi: 10.1136/jitc-2021-002709

Supplementary Figure 6

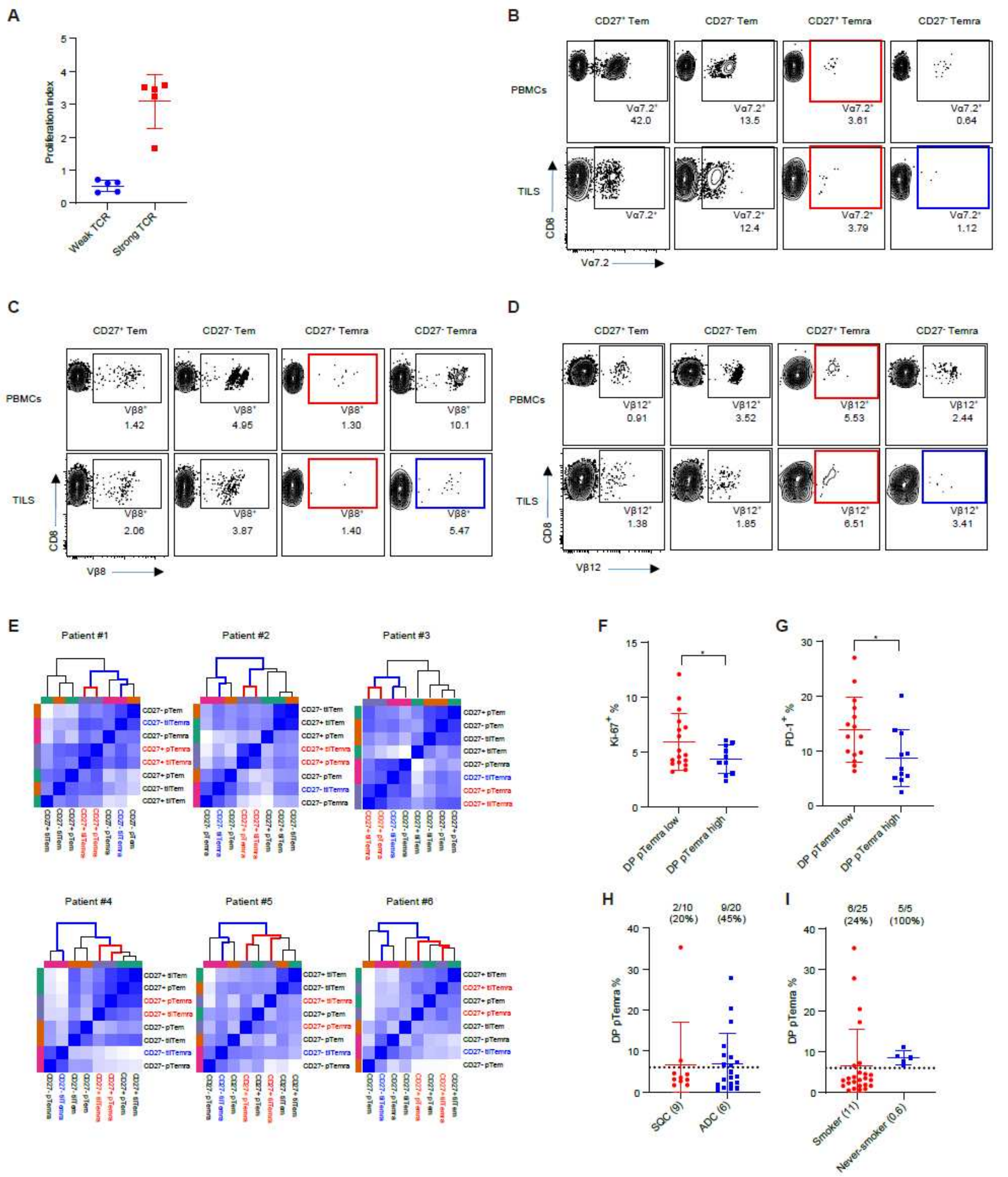

Figure. S6 Correlation between the proportion of DP pTemra and tumor immunogenicity. (A) Tn was purified and labeled with CTV, then stimulated with weak TCR (anti-CD3 (1 µg/ml) and anti-CD28 (0.4 µg/ml)) or with strong TCR (anti-CD3 (5 µg/ml) and anti-CD28 (2 µg/ml)) for 7 days. Proliferated cells were assessed with flow cytometry. (n=5) (B-E) TCR α/β usages

were analyzed from peripheral or tumor infiltrated CD27$^{+/-}$ Tem and CD27$^{+/-}$ Temra cells. Representative data of (B) Vα7.2, (C) Vβ8, and (D) Vβ12 usages. (E) Sample distance matrix were generated using 8 TCR α/β usages (Va2, Vα7.2, Vα12.1, Vβ3, Vβ5b, Vβ8, Vβ12, Vβ13.1). Each matrix includes T cell subsets from one individual. Color coded with blue for close samples and white for distant samples. In the hierarchical clustering above each matrix, red line indicates distance between CD27^{+} tilTemra and CD27^{+} pTemra, blue line indicates distance between CD27^{-} tilTemra and CD27^{+} tilTemra/CD27^{+} pTemra. (F and G) Blood of NSCLC patients were harvested and assessed for (F) Ki-67 and (G) PD-1 expressing CD8^{+} T cells. (H and I) Proportion of DP pTemra in (H) SCC and non-SCC patients or in (I) smoker and never-smoker patients. Dotted line represents ‘DP pTemra % = 6%’. (n=30) (A-I) Data show mean ± SD. Statistical significance was performed with unpaired Student’s t-test.

SUPPLEMENTARY TABLE

Supplementary Table 1

Age		67(40~78)
Sex	Male	17 (65%)
	Female	9 (35%)
Histology	ADC	13 (50%)
	SQC	5 (15%)
	Unknown	8 (35%)
P Stage	IA1	1 (4%)
	IA2	1 (4%)
	IA3	5 (19%)
	Ib	6 (23%)
	IIa	1 (4%)
	IIb	2 (8%)
	IIIa	2 (8%)
	Unknown	8 (31%)
N Stage	N0	8 (31%)
	N1	2 (8%)
	N2	2 (8%)
	Nx	6 (23%)
	Unknown	8 (31%)
M Stage	M0	18 (69%)
	Unknown	8 (31%)

Table. S1 Tumor and blood were harvested from 26 NSCLC patients and used for this study (Figure 1-6).

Supplementary Table 2

Age		67 (44~82)
Sex	Male	23 (77%)
	Female	7 (23%)
Smoking	Current/Former	25 (83%)
	Never	5 (17%)
Histology	ADC	18 (60%)
	SQC	10 (33%)
	NSCLC	2 (7%)
Stage	3A	1 (3%)
	3B	2 (7%)
	4A	14 (47%)
	4B	13 (43%)
EGFR Mutation	Wild	21 (70%)
	E19del	2 (7%)
	L858R	2 (7%)
	Unknown	5 (16%)
PD-L1 expression on Tumor	>50%	16 (53%)
	<50%	13 (43%)
	Unknown	1 (3%)
ICIs	Tecentriq	15 (50%)
	Keytruda	15 (50%)

Table. S2 Blood was harvested from 30 NSCLC patients and used for Figure 6F-G and supplemental Figure 6B-E.
